# Colchicine Is Safe Though Ineffective in the Treatment of Severe COVID-19: a Randomized Clinical Trial (COLCHIVID)

**DOI:** 10.1007/s11606-021-07203-8

**Published:** 2021-11-09

**Authors:** Abdiel Absalón-Aguilar, Marina Rull-Gabayet, Alfredo Pérez-Fragoso, Nancy R. Mejía-Domínguez, Carlos Núñez-Álvarez, David Kershenobich-Stalnikowitz, José Sifuentes-Osornio, Alfredo Ponce-de-León, Fernanda González-Lara, Eduardo Martín-Nares, Sharon Montesinos-Ramírez, Martha Ramírez-Alemón, Pamela Ramírez-Rangel, Manlio F. Márquez, Juan Carlos Plata-Corona, Guillermo Juárez-Vega, Diana Gómez-Martín, Jiram Torres-Ruiz

**Affiliations:** 1 Department of Immunology and Rheumatology, Instituto Nacional de Ciencias Médicas Y Nutrición Salvador Zubirán, Vasco de Quiroga 15, Belisario Domínguez Sección XVI, Tlalpan, Mexico City, Mexico; 2 Bioinformatics, Biostatistics and Computational Biology Unit, Red de apoyo a la investigación Coordinación de Investigación Científica, Universidad Nacional Autónoma de México, Mexico City, Mexico; 3 Department of Gastroenterology, Instituto Nacional de Ciencias Médicas Y Nutrición Salvador Zubirán, Mexico City, Mexico; 4 Department of Infectious Diseases, Instituto Nacional de Ciencias Médicas Y Nutrición Salvador Zubirán, Mexico City, Mexico; 5 Department of Cardiology, Instituto Nacional de Cardiología Ignacio Chávez, Mexico City, Mexico; 6 Department of Clinical Investigation, Instituto Nacional de Cardiología Ignacio Chávez, Mexico City, Mexico; 7 Flow Cytometry Unit, Red de Apoyo a La Investigacion, Coordinacion de Investigacion Cientifica, Universidad Nacional Autonoma de Mexico, Mexico City, Mexico

## Abstract

**Background:**

Colchicine is an available, safe, and effective anti-inflammatory drug and has been suggested as a COVID-19 treatment, but its usefulness in hospitalized severe COVID-19 patients has not been thoroughly demonstrated.

**Objective:**

To address the safety and efficacy of colchicine in hospitalized patients with severe COVID-19.

**Design:**

We conducted a triple-blind parallel non-stratified placebo-controlled clinical trial.

**Participants:**

We recruited 116 hospitalized patients with severe COVID-19 in Mexico.

**Interventions:**

Patients were randomized to receive 1.5 mg of colchicine or placebo at the time of the recruitment in the study (baseline) and 0.5 mg BID PO to complete 10 days of treatment.

**Main Measures:**

The primary composite outcome was the progression to critical disease or death. Besides, we evaluated immunological features at baseline and after recovery or disease progression in 20 patients.

**Key Results:**

Fifty-six patients were allocated to colchicine and 60 patients received placebo. The study was suspended after the second interim analysis demonstrated colchicine had no effect on the primary outcome (OR 0.83, 95%CI 0.35–1.93, *P* = 0.67), nor in the days of ICU and hospital stays. Adverse events were similar between groups (OR 1.63, 95% CI 0.66–3.88, *P* = 0.37). After colchicine treatment, patients had higher BUN and lower serum levels of IL-8, IL-12p70, and IL-17A.

**Conclusions:**

Colchicine is safe but not effective in the treatment of severe COVID-19.

**Trial Registration:**

ClinicalTrials.gov Identifier: NCT04367168.

## INTRODUCTION

The coronavirus disease 2019 (COVID-19) has overwhelmed most health systems around the world since its emergence in December 2019. In Mexico, COVID-19 care centers report up to 45% mortality,^1^ with an urgent need of evidence-based effective therapies. Neutrophils and macrophages are the main cells involved in the pathogenesis of respiratory viral infections. Neutrophilia at COVID-19 diagnosis is a risk factor for critical disease^2^ and neutrophil extracellular neutrophil traps (NETs) are a key pathogenic factor in COVID-19.^3^ Furthermore, NETs contribute to the cytokine storm by promoting IL-1β secretion in macrophages via the inflammasome activation, which induces IL-6 production^4^ and favors a hypercoagulability state.^5^

Currently, there are only few effective treatments to avoid the potentially devastating effects of this virus.^6^ Disease severity and lack of treatment options for COVID-19 have led to off-label prescription of a myriad of drugs based on low-evidence studies. Colchicine is a readily available drug which suppresses the excessive function of neutrophils, monocytes, and macrophages.^7^ Colchicine can inhibit the viroporin E-mediated activation of the inflammasome during SARS-CoV-2 infection,^8^ with the consequent impairment of the production of IL-1β, which leads to an abolishment of the secretion of IL-6 and TNF-α and a decreased recruitment of neutrophils and macrophages.^7^ Colchicine also reduces the production of reactive oxygen species (ROS)^9^ and α-defensin.^7^ Colchicine inhibits neutrophil-platelet aggregation and augments the levels of protein C,^10^ highlighting its anti-thrombotic properties^7^. In addition, colchicine is anti-fibrotic and cardioprotective^11^ and showed to have anti-viral effects in Zika infections.^9^ The safety profile of colchicine at a dose of 0.5–2 mg dose per day has been proved by decades of observational studies and clinical practice.^12^ Therefore, because of its beneficial effects, security profile, and availability, colchicine seemed to be a candidate for COVID-19 treatment. Previous meta-analyses demonstrated a benefit of colchicine treatment in the composite outcome of progression to critical COVID-19 and death.^13–16^ Nonetheless, the benefit of colchicine in hospitalized patients with COVID-19 is uncertain. The pandemic is far from ending and there is an urgency to give new evidence regarding potential treatments for COVID-19. Therefore, the aim of this clinical trial was to address the safety and efficacy of colchicine for the treatment of hospitalized patients with severe COVID-19.

## METHODS

We conducted a parallel triple-blind placebo-controlled clinical trial with a non-stratified 1:1 allocation ratio. A third-party person not involved in the conduction of the protocol was responsible for the randomization of patients using sequentially numbered containers. The same person was responsible for filling and labeling the pill containers either with colchicine or placebo. The study was conducted at Instituto Nacional de Ciencias Médicas y Nutrición Salvador Zubirán and at Instituto Nacional de Cardiología Ignacio Chávez, which are both reference care centers for COVID-19 patients. The recruitment period was from May 2020 to April 2021. The protocol was approved by both institutions’ Investigation and Research Committees and all patients signed informed consent. The register number for this trial is NCT04367168 (COLCHIVID study). We recruited 116 hospitalized adult patients aged 18 to 70 years who tested positive for at least one of the following COVID-19 diagnostic assays: polymerase chain reaction (PCR) for SARS-CoV-2 in nasopharyngeal swab, rapid antigen test, or serum anti-SARS-CoV-2 IgG antibodies.^17^ All patients were classified as severe COVID-19 according to the following definition: respiratory failure, respiratory rate ≥ 30 bpm, oxygen saturation (SpO2) ≤ 93% at rest, PaO2/FiO2 ≤ 300 mmHg.^18^ Due to the risk of toxicity, subjects over 70 years old, those with chronic liver or kidney failure, those who were pregnant, those with puerperium, or those receiving any drug with known interaction with colchicine were excluded. We also excluded patients treated with antimalarial drugs, azithromycin, convalescent plasma, remdesivir, tocilizumab, or baricitinib. Patients under dexamethasone treatment were included since this was the standard of care after the RECOVERY study.^19^ Patients were randomized to receive 1.5 mg of colchicine or placebo PO at baseline, which corresponded to the day of the patient’s recruitment in the study, and, then, 0.5 mg PO BID for 10 days. Placebo tablets were manufactured to be the same size, shape, and appearance as colchicine. The primary composite outcome was evaluated throughout the 10 days of treatment and consisted in death or progression to critical disease, defined as multiple organ failure, shock, or need for invasive mechanical ventilation.^18^ Treatment was suspended in patients who met the primary outcome since most of them presented multiple organ failure and some were not able to keep taking oral drugs.

The secondary outcomes were the duration of intensive care unit (ICU) stay, the total length of hospital admission, the type and number of adverse events, and the changes in the following parameters: vital signs (temperature, respiratory and heart rates, SpO2), inflammatory, and coagulation markers (leukocytes, neutrophil/lymphocyte ratio, C-reactive protein, lactate dehydrogenase, D-dimer and fibrinogen). These features were evaluated at baseline and after 5 days of treatment.

Additionally, as an exploratory study, at baseline and at the time of disease progression or convalescence, we evaluated the following parameters in a subgroup of 20 patients:

- Evaluation of the helper (Th) and cytotoxic (Tc) T cell subsets.
- Assessment of serum circulating NETs, cytokines and chemokines.

Details of our methods are provided in the appendix.

We calculated a sample size of 174 patients to detect a 50% decrease in the incidence of the composite primary outcome, with a power of 80% and a significant value of 0.05. The sample size was calculated using the R project software (R Core Team (2021), R: A language and environment for statistical computing. R Foundation for Statistical Computing, Vienna, Austria. URL http://www.R-project.org/). The researchers in charge of recruitment (AAA, EMN, JTR, MRG, PRR, JCPC), the patients and their treating physicians, and the statistician in charge of the statistical analysis (NRMD) were blinded for the treatment arm throughout the whole study. We performed an intention-to-treat analysis. An independent committee of experts in infectious diseases, Bioethics and Pharmacovigilance carried out two interim analyses throughout the study to protect the safety of the patients. The first interim analysis was made after recruitment of 30% of the calculated sample size to verify the safety of the intervention. After recruitment of 60% of the original sample size, the results from the second interim analysis did not show differences between the treatment arms regarding the primary composite outcome.

### Statistical Analysis

Imputation of missing data was performed using a random forest algorithm in a data matrix. Quantitative variables were expressed as medians and interquartile ranges (IQR). We calculated the odds ratios (OR) with 95% confidence interval (95% CI) for qualitative outcomes. To address the effect of placebo and colchicine treatment on the outcomes, a generalized linear mixed model adjusting for the use of dexamethasone as a random factor was performed. For dichotomic outcomes, we handled error with a binomial distribution, and for quantitative outcomes, the error was handled with Poisson’s distribution. To address the effect of the treatment arm on the secondary outcomes, we calculated the delta of temperature, respiratory and heart rates, SpO2, leukocytes, neutrophil/lymphocyte ratio, C-reactive protein, lactate dehydrogenase, D-dimer, and fibrinogen in each group and compared them with the Mann–Whitney *U* test. The paired sample analysis was also controlled for dexamethasone treatment as a random factor using a generalized linear mixed model. To analyze the treatment effect on the time to develop the primary outcome and on the total length of hospital stay, we used a Cox proportional hazards regression model and calculated hazard ratios (HR) with 95% CI. The statistical analysis was performed using the R project software (R Core Team (2021), R: A language and environment for statistical computing. R Foundation for Statistical Computing, Vienna, Austria. URL http://www.R-project.org/).

## RESULTS

**Fig. 1:**
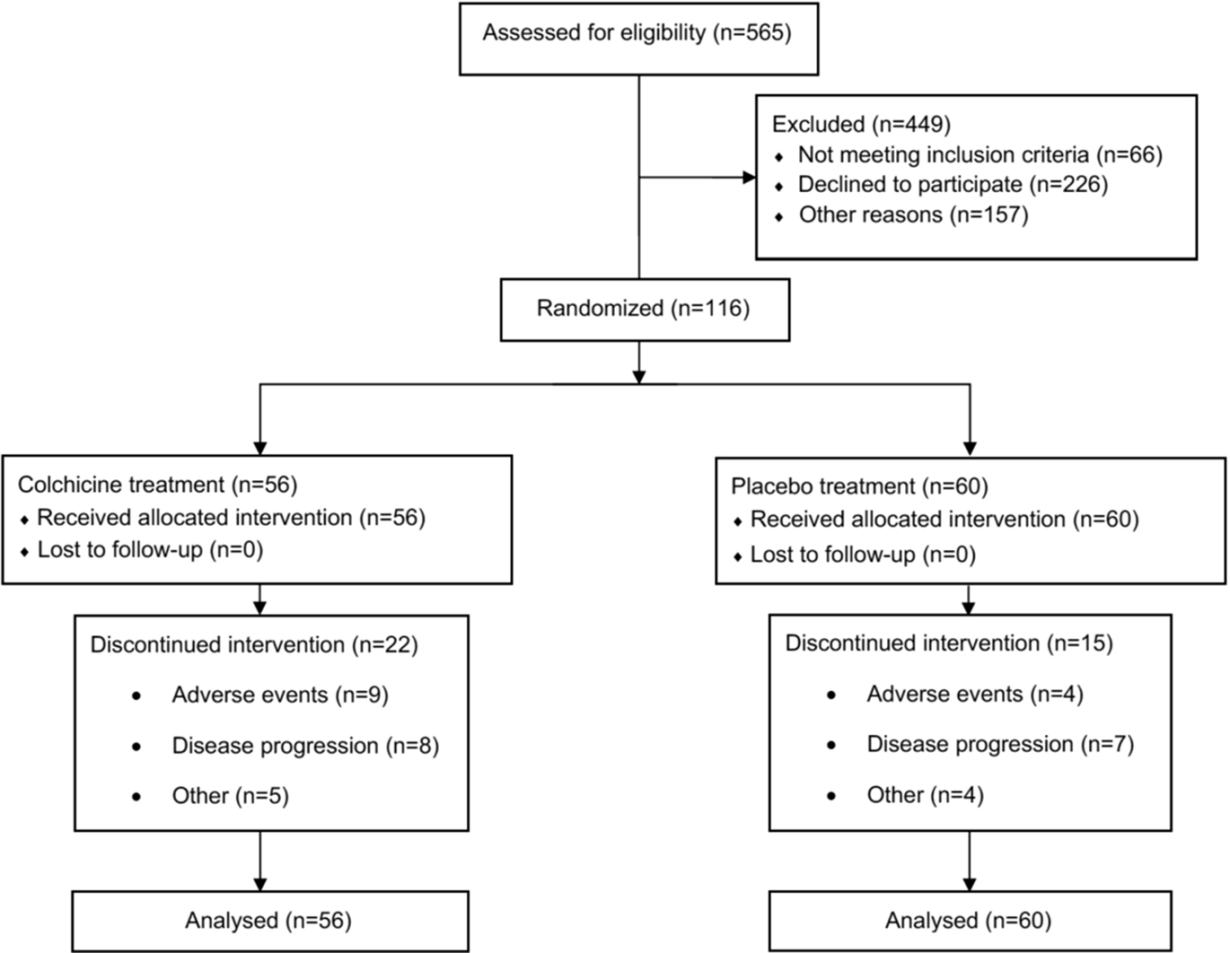
Flow diagram depicting the recruitment and follow-up process throughout the protocol

A flow diagram of the recruitment process is shown in Fig. 1. Fifty-six patients were allocated to receive colchicine treatment and 60 received placebo. The median (IQR) of treatment duration was 10 (3–10) vs 10 (8.5–10) days (*P* = 0.07) in the colchicine and placebo groups, respectively. An intention-to-treat analysis was performed, in which all subjects that received at least one dose of colchicine completed follow-up and were included in the final analysis. Seventy-five percent in the placebo group and 60% of patients in the colchicine group completed 10 days of treatment (*P* = 0.11). After the second interim analysis, the committee informed the research group that although the treatment was safe, it was ineffective to prevent the development of the primary outcome and we decided to suspend the trial.

In Table 1, we depict the baseline clinical features of the patients. Hypertension was more prevalent in the placebo group (20 (33.3%) vs 9 (16.1%), *P* = 0.035). Subjects in the placebo group received dexamethasone (27 (45%) vs 13 (23.2%), *P* = 0.019) more frequently than those in the colchicine group.

**Table 1:** Clinical Features of Patients with Severe COVID-19 According to the Treatment Arm

	Total (*N* = 116)	Placebo (*N* = 60)	Colchicine (*N* = 56)	*P*
Male, *n* (%)	76 (65.5)	39 (65)	37 (66)	0.95
Age, median (IQR)	53 (44–62)	52 (44–62)	55 (43–62)	0.94
Overweight, *n* (%)	53 (46.5)	29 (49.2)	24 (42.9)	0.74
Obesity, *n* (%)	40 (35.1)	22 (36.7)	18 (32.1)	0.69
Type 2 diabetes, *n* (%)	24 (20.7)	13 (21.7)	11 (19.6)	0.82
Hypertension, *n* (%)	29 (25)	20 (33.3)	9 (16.1)	0.03
Cerebrovascular accident, *n* (%)	1 (0.9)	0 (0)	1 (1.8)	0.48
Asthma, *n* (%)	3 (2.6)	2 (3.3)	1 (1.8)	1.00
Chronic obstructive pulmonary disease, *n* (%)	1 (0.9)	0 (0)	1 (1.8)	1.00
Smoking, *n* (%)	28 (24.1)	13 (21.7)	15 (26.8)	0.66
Other, *n* (%)	24 (20.6)	11 (18.3)	13 (23.2)	0.48
Treatment at recruitment				
Non-steroidal anti-inflammatory drugs, *n* (%)	31 (26.7)	16 (26.7)	15 (26.8)	1.00
Antibiotics, *n* (%)	65 (56)	32 (53.3)	33 (58.9)	0.47
Oseltamivir, *n* (%)	18 (15.5)	8 (13.3)	10 (17.9)	0.43
Ivermectin, *n* (%)	26 (23.4)	15 (25)	11 (19.6)	0.50
Dexamethasone, *n* (%)	40 (34.8)	27 (45)	13 (23.2)	0.01
Anticoagulation, *n* (%)	18 (15.5)	11 (18.3)	7 (12.5)	0.44
Symptoms at recruitment				
Cough, *n* (%)	93 (80.2)	49 (81.7)	44 (78.6)	0.81
Headache,* n* (%)	61 (52.6)	34 (56.7)	27 (48.2)	0.45
Fever, *n* (%)	85 (73.3)	45 (75)	40 (71.4)	0.68
Shortness of breath, *n* (%)	92 (79.3)	52 (86.7)	40 (71.4)	0.06
Althralgia, *n* (%)	54 (46.6)	27 (45)	27 (48.2)	0.85
Myalgia, *n* (%)	63 (54.3)	34 (56.7)	29 (51.8)	0.70
Expectoration, *n* (%)	13 (11.2)	6 (10)	7 (12.5)	0.77
Sore throat, *n* (%)	25 (21.6)	15 (25.4)	10 (17.9)	0.37
Rinorrhea, *n* (%)	20 (17.2)	9 (15)	11 (19.6)	0.62
Conjunctivitis, *n* (%)	4 (3.4)	3 (5)	1 (1.8)	0.61
Chest pain, *n* (%)	29 (25)	12 (20)	17 (30.4)	0.20
Vomiting, *n* (%)	6 (5.2)	4 (6.7)	2 (3.6)	0.68
Diarrhea, *n* (%)	31 (26.7)	18 (30)	13 (23.2)	0.52
Fatigue, *n* (%)	38 (32.8)	19 (31.7)	19 (33.9)	0.84
Dysgeusia, *n* (%)	24 (20.6)	14 (23.3)	10 (17.8)	0.52
Anosmia, *n* (%)	25 (21.5)	15 (25)	10 (17.8)	0.37
Nutri-CoV score				
Very high risk, *n* (%)	27 (23.3)	14 (23.3)	13 (23.2)	0.98
High risk, *n* (%)	50 (43.1)	23 (38.3)	27 (48.2)	0.28
Moderate risk, *n* (%)	25 (21.1)	15 (25)	10 (17.2)	0.35
Low risk, *n* (%)	14 (11.8)	8 (13.3)	6 (10.3)	0.66
Oxygen therapy (lts/min)				
1–5 lts/min	89 (76.7)	49 (81.7)	40 (71.4)	0.28
6–15 lts/min	27 (23.3)	11 (18.3)	16 (28.5)	0.19

**Fig. 2:**
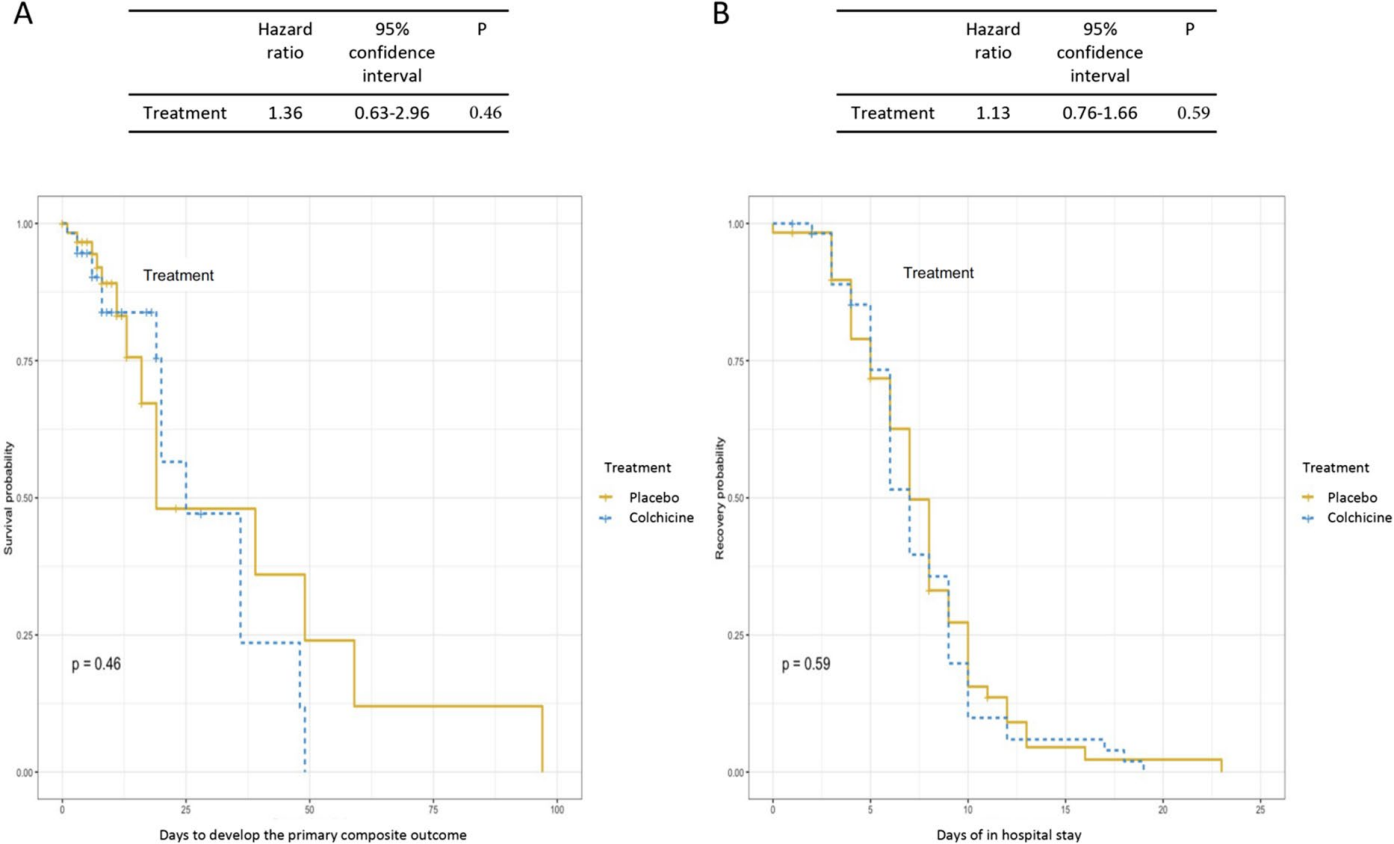
Kaplan–Meier curves showing the time for the development of the primary outcome (**A**) and the total amount of days of hospital stay (**B**)

The baseline laboratory and radiologic features are depicted in Table 2. Four patients died in the colchicine treatment arm (4/56, 7.14%) and six in the placebo group (6/60, 10%) (OR 0.69 (95% CI 0.21–2.55), *P* = 0.74). After adjustment for dexamethasone use and hypertension diagnosis, colchicine treatment had no effect on the primary outcome (OR 0.83 (95% CI 0.35–1.93), *P* = 0.67). There was no difference in the length of ICU stay (0 (0–0.75) vs 0 (0–1), *P* = 0.29), nor in the number of days of hospital stay (8 (5–10.75) vs 7.5 (6–11.5), *P* = 0.73). Figure 2 shows the survival curves for the composite outcome and the probability of recovery, with no difference between groups.

**Table 2:** Laboratory and Radiologic Features of the Patients with Severe COVID-19

	Total (*N* = 116)	Placebo (*N* = 60)	Colchicine (*N* = 56)	*P*
Leukocytes, median (IQR), × 10^3^ cells/µL	8.05 (6–12.3)	7.65 (5.65–12.25)	8.15 (5.9–12.5)	0.45
Lymphocytes, median (IQR), cells/µL	718.4 (422.7–1013.2)	674 (422.7–985)	746.2 (432.5–1118.5)	0.46
Neutrophils, median (IQR), cells/µL	6380.6 (4502.6–11,167.6)	6318.4 (4269.9–10,838.6)	6439.8 (4540.2–11,236.2)	0.54
Neutrophil/lymphocyte ratio, median (IQR)	10.9 (5.6–18.6)	10.2 (4.7–17.9)	10.9 (5.5–18.6)	0.49
Platelets, median (IQR), cells/µL	225,500 (159,000–306,000)	236,000 (168,500–297,000)	214,000 (130,000–306,000)	0.38
Creatinine, median (IQR), mg/dL	0.86 (0.72–1.05)	0.86 (0.72–0.86)	0.86 (0.73–0.86)	0.58
ALT, median (IQR), UI/L	35.9 (22.5–60)	40.6 (25.3–67.7)	34 (21.7–47.45)	0.22
AST, median (IQR), UI/L	39 (26.5–53)	40.5 (29.1–62.4)	37.7 (25.3–45.5)	0.06
Albumin, median (IQR), g/dL	3.73 (3.46–4.01)	3.79 (3.48–4.07)	3.70 (3.44–3.96)	0.12
Globulins, median (IQR) g/dL	3.18 (2.84–3.39)	3.11 (2.82–3.33)	3.24 (2.87–3.57)	0.20
C-reactive protein, median (IQR), mg/dL	13.9 (7.13–20.6)	13.28 (5.7–19.2)	15.06 (7.53–26.8)	0.39
Ferritin, median (IQR), ng/dL	616.5 (327.6–974.8)	545.2 (249.1–1028.7)	673.1 (347.2–974.7)	0.85
Lactic dehydrogenase, median (IQR), IU/L	331 (276.5–406.5)	341 (292–401)	308 (269–407)	0.57
Creatine phosphokinase, median (IQR), U/L	88 (46.6–153.9)	87 (52–155)	91 (43–152)	0.90
D-dimer, median (IQR), ng/mL	649 (389.5–1095.5)	627 (401–1085)	701 (383–1100)	0.70
Troponin I, median (IQR), pg/mL	4.3 (3.15–6.2)	4.3 (3–6.1)	4.3 (3.4–6.6)	0.61
Fibrinogen, median (IQR), mg/dL	655 (562–763.5)	633 (498–774)	642 (605–763)	0.68
Thoracic computed tomography involvement				
> 50%, *n* (%)	71 (61.2)	40 (66.7)	31 (55.3)	0.21
20–50%, *n* (%)	38 (32.2)	15 (25)	23 (39.7)	0.06
< 20%, *n* (%)	7 (5.9)	5 (8.3)	2 (3.4)	0.28

Table 3 depicts the main adverse events observed throughout the study. Colchicine treatment was related to a higher prevalence of adverse effects (15 (26.8%) vs 7 (11.7%), *P* = 0.057), although the difference was not statistically significant (OR 1.63 (95% CI 0.66–3.88), *P* = 0.37). Gastrointestinal adverse effects were the most frequent and led to suspension of treatment in 8 (53.3%) vs 4 (57.1%) patients in the colchicine and placebo groups, respectively (OR 1.167 (0.21–5.89), *P* = 0.86). There were no renal or hematological adverse events.

**Table 3:** Adverse Events Observed Throughout the Study According to the Treatment Group

	Total (*N* = 116)	Placebo (*N* = 60)	Colchicine (*N* = 56)	*P*
Adverse events, *n* (%)	22 (19)	7 (11.7)	15 (26.8)	0.057
Gastrointestinal, *n* (%)	19 (16.4)	7 (11.7)	12 (21.4)	0.210
Diarrhea, *n* (%)	12 (10.3)	4 (6.7)	8 (14.3)	0.228
Abdominal pain,* n* (%)	4 (3.4)	1 (1.7)	3 (5.4)	0.352
Nausea, *n* (%)	3 (2.6)	2 (3.3)	1 (1.8)	1.00
Singult, *n* (%)	1 (0.9)	1 (1.7)	0 (0)	1.00
Hypertransaminasemia, *n* (%)	5 (4.3)	2 (3.3)	3 (5.4)	0.671
Other, *n* (%)	2 (1.7)	1 (1.7)	1 (1.8)	1.00

**Fig. 3:**
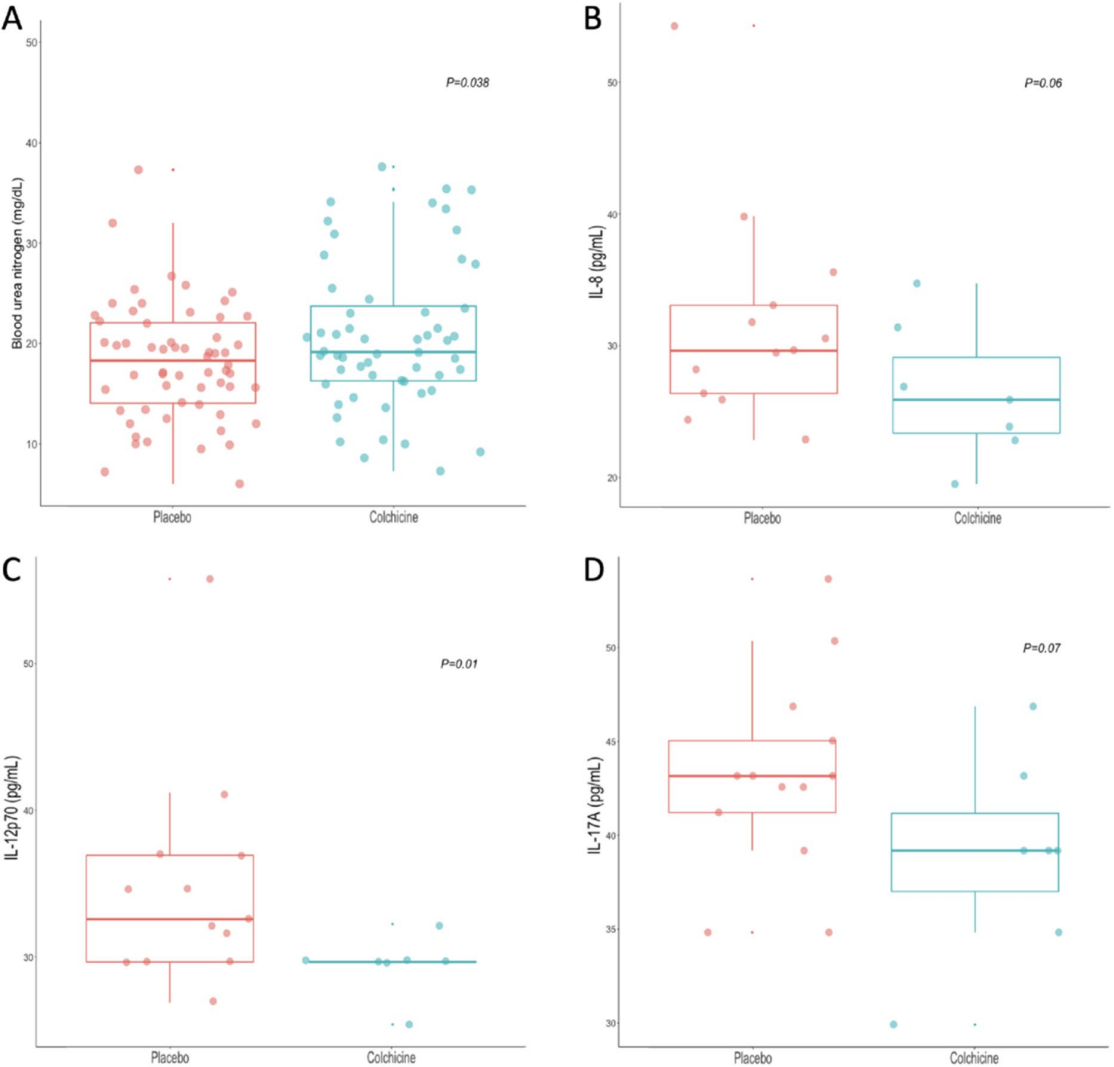
Analysis of the laboratory and immunological features of patients with severe COVID-19 after treatment. Patients who received colchicine had a higher BUN (**A**), and lower levels of IL-8, IL-12p70, and IL-17A (**B**–**D**)

Colchicine treatment had no effect on vital signs (temperature, respiratory and heart rates, or SpO2) nor in the inflammatory parameters. In Table 4, we depict the analysis of the secondary outcomes according to the treatment arm. Patients who received colchicine had a higher BUN (20.6 mg/dL (16.3–27.9) vs 18.7 (13.3–22.7), *P* = 0.038) after treatment (Fig. 3).

**Table 4:** Analysis of the Clinical and Laboratory Features Evaluated as Secondary Outcomes According to the Treatment Arm

	Colchicine *N* = 56 Δ Median (IQR)	Placebo *N* = 60 Δ Median (IQR)	*P*
Temperature (°C)	0.20 (− 0.15 to 0.50)	0.30 (− 0.2 to 0.82)	0.45
Respiratory rate (breaths per minute)	1.00 (− 1.00 to 6.25)	2.00 (− 1.00 to 6.00)	0.90
Heart rate (beats per minute)	16 (0.75 to 27.00)	15.5 (3.00 to 31.25)	0.69
SpO2 (%)	− 4 (− 0.90 to 1.00)	− 5 (− 11.00 to 57.70)	0.84
Leukocytes (× 10^3^ cells/µL)	0.50 (− 1.55 to 2.05)	− 0.10 (− 1.87 to 7.90)	0.68
Lymphocytes (cells/µL)	− 177.20 (− 498.80 to 53.35)	− 132.60 (− 509.10 to 121.6)	0.98
Neutrophil/lymphocyte ratio	0.77 (− 1.43 to 6.79)	2.66 (− 0.39 to 10.12)	0.09
C-reactive protein (mg/dL)	4.30 (0.68 to 9.61)	4.38 (0.00 to 10.29)	0.89
Lactic dehydrogenase (IU/L)	34 (− 9.00 to 87.25)	65 (0.6 to 111.60)	0.12
D-dimer (ng/mL)	− 109.5 (− 823.00 to 135.30)	− 19.50 (− 277.00 to 140.30)	0.18
Fibrinogen (mg/dL)	24 (− 0.30 to 137.00)	0.00 (− 21.00 to 135.5)	0.41

The immunologic parameters evaluated in the 20 patients who participated in the exploratory study are depicted in Table 5. Colchicine had no effect on the proportion of T cell subsets and NETs.

**Table 5:** Immunological Features of COVID-19 Patients at Baseline and After Treatment

Variable	Pre-treatment Median (IQR) *N* = 20	Post-treatment Median (IQR) *N* = 20	*P*
Serum cytokines and chemokines			
IL-1b (pg/mL)	4.23 (3.92–5.16)	4.23 (3.27–4.55)	0.31
IL-2 (pg/mL)	36.78 (33.79–42.17)	33.26 (30.50–37.25)	0.02
IL-4 (pg/mL)	3.15 (3.15–3.58)	3.15 (2.64–3.25)	0.03
IL-5 (pg/mL)	188.90 (163–206.7)	161.30 (156–177.10)	0.003
IL-6 (pg/mL)	16.49 (13.57–25.14)	14.11 (9.45–15.43)	0.30
IL-7 (pg/mL)	52.90 (44.30–60.79)	44.30 (34.62–48.71)	0.94
IL-8 (pg/mL)	33.49 (27.80–38.69)	27.80 (24.28–33.49)	0.03
IL-10 (pg/mL)	27.56 (24.55–31.90)	24.55 (20.40–25.87)	0.004
IL-12 (p70) (pg/mL)	34.65 (32.24–39.11)	29.67 (29.67–35.22)	0.012
IL-13 (pg/mL)	4.64 (4.64–5.37)	5.37 (4.64–5.78)	0.47
IL-17 (pg/mL)	43.16 (39.18–46.87)	42.19 (38.09–45.50)	0.11
G-CSF (pg/mL)	114.90 (102.5–139.70)	99.82 (90.26–108.8)	0.002
GM-CSF (pg/mL)	9.99 (8.91–10.88)	8.62 (8.02–9.73)	0.001
IFNγ (pg/mL)	16.65 (13.85–19.40)	14.89 (13.63–16.65)	0.09
MCP-1 (MCAF) (pg/mL)	72.82 (52.80–108)	64.39 (48.79–84.05)	0.26
MIP-1b (pg/mL)	52.02 (40.81–67.66)	44.12 (39.23–55.20)	0.15
TNFa (pg/mL)	136.3 (124.20–144.10)	116.7 (104.6–133.3)	0.004
Serum neutrophil extracellular traps			
NETS (ODI)	1.13 (1.06–1.22)	1.09 (1.03–1.17)	0.240
Cell subsets			
T CD4^+^ Th1 (%)	19.95 (11.55–30.48)	32.45 (20.45–46.13)	0.06
T CD4^+^ Th2 (%)	17.25 (12.30–24.88)	25 (16.45–28.35)	0.08
T CD4^+^ Th17 (%)	1.88 (0.78–4.03)	5.40 (0.76–8.12)	0.31
T CD8^+^ Tc1 (%)	22 (11.15–35.68)	33.45 (22.95–47.10)	0.09
T CD8^+^ Tc2 (%)	31.65 (24.48–46.73)	31.65 (24.55–42.20)	0.93
T CD8^+^ Tc17 (%)	0.3 (0.10–1.41)	0.19 (0.19–1.09)	0.85

After adjusting for dexamethasone treatment, patients who received colchicine had lower levels of serum IL12p70 (29.67 (29.67–29.67) vs 39.1 (34.8–43.1), *P* = 0.01), and a trend towards lower serum levels of IL-8 (25.9 (822.8–31.38) vs 29.6 (25.9–35.5), *P* = 0.06), and IL-17A (39.1 (34.8–43.1) vs 43.1 (39.1–46.8), *P* = 0.07) (Fig. 3).

## DISCUSSION

Our study found that colchicine treatment is safe, but ineffective in preventing disease progression or death in patients with severe COVID-19. The role of colchicine in the treatment of COVID-19 has been evaluated in many clinical scenarios. Among ambulatory patients, the COLCORONA study tested a composite outcome of death and hospitalization.^20^ They found that colchicine reduced their primary outcome (OR: 0.75, 95% CI 0.57–0.99), though did not reduce hospitalization. In both ambulatory and hospitalized patients, a retrospective case–control study found decreased mortality among 70 patients who took colchicine compared to controls (HR 0.24 (95% CI 0.09–0.67)).^21^ Colchicine also improved C-reactive protein, as well as the lymphocyte count and the neutrophil/lymphocyte ratio.^21^

An open study of 72 patients showed that colchicine decreased oxygen requirement days (4 vs 7 days) and hospital stay (7 vs 9 days).^22^ Sandhu et al. performed a retrospective case–control study in which they evaluated 53 patients treated with colchicine and compared them with 144 subjects under standard treatment.^23^ Patients who received colchicine were less frequently intubated and had lower mortality rate and a higher probability of being discharged from the hospital. However, the control group had a higher incidence of T2DM, hypertension, and chronic kidney disease.^23^ The GRECCO-19 study was an open-label, randomized study in which colchicine was added to the standard medical treatment.^24^ Colchicine reduced the likelihood of clinical deterioration (OR 0.11 (95% CI 0.01–0.96), *P* = 0.046), with higher cumulative survival (83 vs 97%, *P* = 0.03).

In an Italian study including 122 patients, colchicine was added to standard treatment (antimalarial drugs, glucocorticoids, and antivirals).^25^ Colchicine treatment was associated with a lower probability of death (HR = 0.151 (95% CI 0.062 to 0.368), *P* < 0.0001).^25^ Colchicine along with the standard treatment has also been tested to avoid the cytokine storm due to COVID-19.^26^ In a case–control study of 66 patients, colchicine was prescribed along multiple interventions including antimalarial drugs, azithromycin, tocilizumab, and remdesivir.^26^ They found that colchicine decreased 28-day mortality, reduced COVID-19 severity, and improved the probability of hospital discharge.^26^

The RECOVERY study randomized 5610 patients to colchicine and 5730 to many different standard treatments; 94% were receiving steroids. The median duration of colchicine treatment was 6 days (IQR 3–9 days). Similar to our study, they found no difference in mortality, time to discharge, or progression to critical disease.^27^

Our study supports the safety of colchicine in the treatment of severe COVID-19, since we did not find a difference in the proportion of adverse events between groups and no hematological or renal severe adverse events were registered. According to previous studies demonstrating changes in kidney function during colchicine treatment,^28^ we found a mild increase of BUN without clinical significance or creatinine elevation.

Regarding the immunologic features, longitudinal studies have shown that high levels of pro-inflammatory cytokines are maintained for over 30 days in patients with severe COVID-19.^29^ IL-8, a biomarker of COVID-19 severity,^30^ has been related to a longer disease duration^31^ and is inversely correlated with the time from admission to death.^32^ Furthermore, high levels of IL-8 are associated with elevated D-dimer and decreased PaO2/FiO2 ratio. This chemokine induces neutrophil degranulation, promoting a pro-thrombotic state.^30^ IL-12p70 and IL-17A are cytokines that remain high in convalescent severe COVID-19 patients and are related to chronic inflammation and long-lasting symptoms.^29^ Besides, elevated IL-17A has been observed in late-stage severe COVID-19 cases^33,34^ and has been related to increased morbidity and mortality.^35^ IL-17A is a pro-inflammatory cytokine related to enhanced endothelial damage and platelet activation leading to thrombosis,^36^ which are fundamental features of severe COVID-19. In patients with atrial fibrillation, it has been shown that colchicine downregulates the activation of IL-17A and renin-angiotensin axes, which may contribute to its anti-fibrotic effect.^37^ In agreement with these results, we found that colchicine promoted a decline in IL-17A levels. Further studies are needed to evaluate whether colchicine-mediated reduction of these cytokines results in COVID-19 lung damage improvement. Regarding the amount of NETs, our results are similar to those found in patients with familial Mediterranean fever^38^ since we observed that colchicine did not affect the production of NETs, which are key drivers of COVID-19 severity.^39^ Our data suggest that the suppression of the neutrophil function and the anti-inflammatory properties of colchicine are not potent enough to counteract the cytokine storm in patients with severe COVID-19. However, colchicine might have an effect to reduce the risk for development post-COVID-19 manifestations; this merits additional clinical research to find out the potential benefit.

The main strength of our study is its triple-blind placebo-controlled design. The close follow-up of patients allowed us to detect adverse events in a timely manner and conclude that colchicine treatment is safe in hospitalized patients with severe COVID-19. Furthermore, our statistical analysis was blinded and controlled for potential confounders, which makes our conclusions more robust. Due to the early termination of the study, our trial has a smaller sample size than originally planned. Nonetheless, with the recruited sample size, the study had sufficient power (95.6%) to detect a diminishment of 50% in the previously described 45% mortality in our COVID-19 centers.^1^ The principal factor affecting recruitment was that patients did not feel comfortable participating in clinical trials at the beginning of the pandemic, because of the lack of knowledge about the safety of the drugs and of the natural history of COVID-19. In addition, many patients received azithromycin and antimalarial drugs for COVID-19, precluding their recruitment.

In conclusion, colchicine treatment in hospitalized patients with severe COVID-19 is safe but not effective for the prevention of disease progression or death. After colchicine treatment, patients had a higher BUN but lower levels of IL-8, IL-12p70, and IL-17. Currently, guidelines for the management of hospitalized adults with COVID-19 recommend against the use of colchicine.^40^ Our study contributes to the reinforcement of this recommendation and will be useful for front-line physicians facing severe COVID-19.

## Appendix methods

- Evaluation of the helper (Th) and cytotoxic (Tc) T cell subsets. 15 mL of peripheral venous blood were drawn and separated by density gradients after centrifugation with Ficoll-paque (GE Healthcare Life Sciences, Illinois, USA) to isolate peripheral blood mononuclear cells (PBMCs). Five million cells were washed twice with PBS and resuspended in 1 mL of RPMI with phenol red supplemented with 10% fetal bovine serum (FBS). Afterwards, the PBMCs were stimulated with 50 ng/mL phorbol myristate acetate (PMA) (MERCK, Kenilworth, New Jersey, USA), 1 μg/mL ionomycin (MERCK, Kenilworth, New Jersey, USA), and monensin (BD Biosciences, Franklin Lakes, New Jersey, USA) for 5 hours in an incubator at 37ºC and 5% CO2. The cells were then washed twice with 5% FBS in PBS and stained with the following fluorochrome coupled antibodies: mouse anti human CD4 Alexa Fluor 488 and mouse anti human CD8 PE-Dazzle 594 (both from BioLegend, San Diego, California, USA). Afterwards, the cells were fixed and permeabilized using the BD cytofix/cytoperm kit (BD Biosciences, Franklin Lakes, New Jersey, USA). The cells were stained with the following fluorochrome coupled antibodies: mouse anti human IFN-γ APC, IL-4 PE and IL-17A BV421 (Biolegend, SanDiego, California, USA). One million events were acquired in a 4 laser LSR Fortessa flow cytometer. The following T cell subsets were defined: Th1 (CD4+, IFN-γ+), Th2 (CD4+, IL-4+), Th17 (CD4+, IL-17A+), Tc1 (CD8+, IFN-γ+), Tc2 (CD8+, IL-4+), Tc17 (CD8+, IL-17A+). The analysis was performed using the Flow-Jo software V10.8.0 (BD Biosciences, Franklin Lakes, New Jersey, USA).
- Assessment of serum circulating NETs, cytokines and chemokines. The serum levels of neutrophil elastase (NE)-DNA complexes were evaluated by sandwich ELISA as previously described.^41^ Briefly, a high binding 96 well ELISA plate was coated overnight (ON) at 4ºC with 1: 2000 mouse anti human NE (Calbiochem, San Diego, California, USA) in PBS. After three washes with 0.05% Tween/PBS (Thermofisher, Waltham, Massachusetts, USA), the non-specific binding sites were blocked with 1% bovine serum albumin (BSA) for six hours. The serum samples were diluted 1:100 in 1% BSA. The plate was washed three times with 0.05% Tween/PBS and 100 μL of the diluted samples were added and incubated ON at 4ºC. After three washes with 0.05% Tween/PBS, the plate was incubated at room temperature (RT) for one hour with 1:100 mouse anti human dsDNA (EMD Millipore Burlington, Massachusetts, USA). Afterwards, the plate was washed three times with 0.05% Tween/PBS and incubated for one hour with 1: 10000 anti mouse HRP (BioRad, Hercules, California, USA) at RT. After five washes with 0.05% Tween/PBS, the 3,3',5,5'-Tetramethylbenzidine (TMB) substrate (Thermofisher, Waltham, Massachusetts, USA) was applied, and the plate was read at 450 nm in an ELISA plate reader after adding stop solution. Additionally, the serum levels of 29 cytokines and chemokines were measured using the MILLIPLEX Multi-Analyte Profiling (MAP) Human Cytokine/Chemokine Magnetic Bead Panel 29-plex kit (EMD Millipore Burlington, Massachusetts, USA).
